# New Provider Models for Sweden and Spain: Public, Private or Non-profit? Comment on "Governance, Government, and the Search for New Provider Models"

**DOI:** 10.15171/ijhpm.2016.87

**Published:** 2016-06-29

**Authors:** Patrick P.T. Jeurissen, Hans Maarse

**Affiliations:** ^1^Radboud Institute for Health Sciences, Celsus Academy for Sustainable Healthcare, and Scientific Institute for Quality of Healthcare, Radboud University Medical Center, Nijmegen, The Netherlands.; ^2^Maastricht University, Maastricht, The Netherlands.

**Keywords:** Privatization, For-Profit Healthcare, Provider Reform, Not-for-Profit Providers

## Abstract

Sweden and Spain experiment with different provider models to reform healthcare provision. Both models have in common that they extend the role of the for-profit sector in healthcare. As the analysis of Saltman and Duran demonstrates, privatisation is an ambiguous and contested strategy that is used for quite different purposes. In our comment, we emphasize that their analysis leaves questions open on the consequences of privatisation for the performance of healthcare and the role of the public sector in healthcare provision. Furthermore, we briefly address the absence of the option of healthcare provision by not-for-profit providers in the privatisation strategy of Sweden and Spain.

## Introduction

Privatization is an ambiguous instrument in the health policy-makers’ toolbox ranging from the outsourcing of operational activities (eg, laundry, security) and joint ventures to the conversion of public hospitals into private ones or the penetration of for-profit providers into an otherwise public healthcare provision system. It is also a contested instrument that almost everywhere provokes a merely ideological debate between proponents and adversaries. In several countries privatisation is introduced (or considered) as an effective antidote to widespread problems in public provider systems such as weak capital investment routines, poor reward structures and work rule arrangements, lack of patient responsiveness client and politically motivated short-term interference. Saltman and Duran^1^ discuss two countries, Sweden and Spain, respectively, where privatisation is currently experimented with, although in Sweden in a more radical way than in Spain. A common characteristic of the governance structure in both countries is the prominent role of the regional level in healthcare provision; they belong to the category of decentralized national health systems and thus their probably exists considerable variety in ownership types between regions in these countries.

The main strength of their article lies in their description of *how* the governance of healthcare provision has been changing over the past decades into the direction of privatisation. From their analysis also follows that privatisation can be shaped in many different ways: privatisation in Sweden is quite different from privatisation in Spain. Unfortunately, they are rather silent on the longer-term consequences of privatisation for healthcare provision. Does the private sector indeed outperform the public sector in terms of efficiency, quality of care (including continuity of care), innovativeness and patient-responsiveness, as the advocates of privatisation claim? What is its impact on healthcare expenditures and, last but not least, on the performance of the public sector itself? One probable reason for not addressing these important questions is the lack of sound empirical analysis in these countries.

## Sweden: Privatization of Primary Care

A distinctive characteristic of Sweden’s system for primary care services is that doctors are unionized employees of the county council. Whereas the system features (very) low volumes in terms of visits per patient et cetera, it scores well in terms of quality of care, also in comparison to many other European countries.^2^ Nevertheless, there were continuing signs of public dissatisfaction on waiting times, the lack of convenient opening times and, perhaps most important, the difficulty of building a personal relationship with a doctor. In order to address these problems, the government implemented a reform in 2010 requiring all 21 counties to allow private primary care practices to be established, and fund those practices on a contract basis, tied to the number of patients they saw. The reform built upon earlier initiatives of 5 counties. The purpose of the reform was not only to increase patient choice but also to foster efficiency and patient responsiveness. The reform had a significant impact on primary care, especially in the main urban areas. Stockholm in many ways has pioneered this trend.^3^ Within eight months, the number of private for-profit centres had increased by more than 20%; and within two years 50% of all primary care visits took place in non-public practices. These are, by all standards, impressive numbers that must have surprised many experts and stakeholders. While the reform seems to have increased public satisfaction, its impact on healthcare expenditures remains unclear in the analysis of Saltman and Duran. In this respect one should keep in mind that greater efficiency does not necessarily mean *lower* total expenditures.

Perhaps unanticipated, it are many new private for-profit firms *from outside* the regular healthcare sector, including risk-capital funds, that operate the majority of these additional care providers. The pace of growth seems breath-taking and resembles the unanticipated increase of for-profit clinics in the United States after the introduction of the Medicare program (1966) or the rapid privatization of the public hospitals in the German Democratic Republic after 1989.^4^ The Swedish experience illustrates the findings of studies that hypothesize that for-profit providers seem more responsive to (new) external incentives than either public or non-profit providers.^5^ Also notice in this respect that earlier reforms to improve the primary care system *from within* had largely failed.

The Swedish reform raises many pertinent questions on privatisation in healthcare. What will be the structural impact of the allowance of private for-profit practice in primary care? Will it imply the eventual end of the public delivery system or will it only add an extra layer to primary care, making the provision system more diverse? Who will benefit from this development and who will incur its costs? How will the penetration of for-profit health centres affect the performance of the public centres? Experience in other countries including the United States and Germany indicate, that the threat of the private sector does enforce the public sector to an extent to adapt their strategy in order to survive in a competitive environment. Finally, there is a perhaps inconvenient lesson for public policy-makers: reforms mobilizing external forces to bring about change may have more rapid and radical effects than reforms aiming to change the system *from within*.

## Spain: Privatization of Hospitals?

The comparison of privatisation in Spain and Swedish is a clear illustration of the ambiguity of the concept of privatisation. Saltman and Duran explain that a radical privatisation policy is inconceivable in the Spanish political context. Therefore, a pragmatic approach was the only politically feasible option. Privatisation is sought by means of administrative concessions to private firms to run a public hospital during a contract period in return for a (performance-related) fee. The hospital remains in public ownership. The reform is driven by the need to reduce public deficits which are closely related to the financial crisis that has severely hit Spain since 2009. This suggests that the Spanish version of privatisation had another objective than the reform in Sweden: to save costs instead of enhancing consumer choice and fostering patient responsiveness. Or: trading negative cash-flows on the public balance sheet for commercial returns on publicly funded assets. Saltman and Duran do not discuss whether the reform has been successful. Instead, they emphasize the contested nature of privatisation in Spanish politics, where the conservative political forces manifest themselves as an advocate of privatisation initiatives whereas the left political forces strongly oppose privatisation. The political debate has been very ideological so far, also because of the absence of any impartial and scientific evaluation. One may wonder, however, whether such an evaluation would put an end to the ideological debate. The results might still be refuted by either the advocates or opponents of privatisation. Nevertheless their effect on public goals such as access, efficiency and quality of care, a secondary analysis of available studies show that public and private hospital providers spend their resources in a different way which illustrates deeply political disputes. Physicians, managers, and shareholders are higher rewarded in private hospitals; nurses and supporting staff are better paid in public hospitals; for-profits have higher capital and administrative expenses.^4^

## Discussion

Privatisation in healthcare (and other public sectors) appears a complex issue. It is not only an ambiguous concept having many different meanings, but also a contested concept almost inevitably eliciting an ideological debate between opponents and advocates. It is particularly the fear for its adverse impacts on both universal access and the employment conditions of the workers in public hospitals that helps to explain the critical stance towards privatisation. Furthermore, there is a widespread fear for loss of public control of the government in healthcare.

The analysis of Saltman and Duran demonstrates another aspect of the complexity of privatisation: it can be used as a policy instrument for a multitude of policy objectives, ranging from increasing patient choice and patient responsiveness to improving efficiency, tapping extra capital resources, and so on. Whereas privatisation in Sweden seems to be mainly demand-driven (choice and responsiveness), privatisation in Spain primarily follows a supply-driven approach (efficiency, cost savings). Unfortunately, Saltman and Duran do not spend much attention to the regulatory arrangements surrounding privatisation in both countries. These arrangements are obviously critical for its success.

As Saltman and Duran rightly spell out, privatisation almost inevitably seems to provoke an ideological debate on the pros and cons of privatisation. Prominent issues in this respect are its consequences for healthcare costs (including administrative costs, access to healthcare, quality of care, efficiency, patient responsiveness, salaries, and work conditions). In our view, a very important aspect of privatisation is its impact on the public-private mix in healthcare provision. For instance, what is the balance between the public and private sector? Is the private sector involved in ‘cherry-picking’? Does the success of the private sector enforce public providers to perform better? Does privatisation take place by take-overs or by the erection of new providers penetrating into the public system? These questions are essential to understand the eventual impact of privatisation on the landscape in healthcare provision.

A noticeable aspect of privatisation in Sweden en Spain is the ‘non-role’ of the private not-for-profit option in the reform (notice that those who define privatisation in terms of for-profit providers would consider this model at best halfway-privatisation). This model has a long history in mainly social insurance systems (‘Bismarck systems’) including for instance Belgium and the Netherlands. In the latter country even *all* hospitals are private not-for-profit entities (there is still a legal ban on for-profit hospital care).^6^ In Germany, not-for-profit hospitals co-exist with public and for-profit hospitals; the not-for-profit sector has also consolidated its traditionally strong position in hospital care. In the Netherlands, two new non-profit provider organisations, one in home care and the other one in caring for patients with Parkinson, are among the most successful organizations in healthcare provision, particularly in terms of patient satisfaction, (administrative) costs and outcomes. There is no clear answer to the question why the not-for-profit model has been left out of consideration. Neither do Saltman and Duran address this question in depth, even though they present the not-for-profit model as an interesting alternative for the public and for-profit model in the beginning of their analysis. It may be that the social and cultural forces which once resulted in the creation of not-for-profit provider organisation in the aforementioned countries – the wish to self-organise healthcare for its constituent community – have largely vanished in modern healthcare, so that the not-for-profit option has no future.

Going beyond Sweden and Spain, it seems hardly unthinkable for the next decades that the private for-profit sector will not extend its role in healthcare provision. The big challenge for governments will be how to design a governance structure which best aligns public and private interests in healthcare.

## Ethical issues

Not applicable.

## Competing interests

Authors declare that they have no competing interests.

## Authors’ contributions

PJ wrote the original manuscript. Both authors contributed equally to the analysis and final manuscript.

## Authors’ affiliations

^1^Radboud Institute for Health Sciences, Celsus Academy for Sustainable Healthcare, and Scientific Institute for Quality of Healthcare, Radboud University Medical Center, Nijmegen, The Netherlands. ^2^Maastricht University, Maastricht, The Netherlands.
